# miR4673 improves fitness profile of neoplastic cells by induction of autophagy

**DOI:** 10.1038/s41419-018-1088-6

**Published:** 2018-10-19

**Authors:** Kağan Dökümcü, Mary Simonian, Ramin M. Farahani

**Affiliations:** 1 Institute of Dental Research, Westmead Institute for Medical Research and Westmead Centre for Oral Health, Westmead, NSW Australia; 2 Department of Life Sciences, The University of Sydney Dental School, Faculty of Medicine and Health, University of Sydney, Sydney, NSW 2006 Australia

## Abstract

Therapeutic resistance of neoplasms is mainly attributed to gradual evolution of mutational profile^1^. Here, we demonstrate a microRNA-mediated mechanism that effectively improves fitness of SKBR3 mammary carcinoma cells by cytoplasmic reprogramming. The reprogramming is triggered by endogenous miR4673 transcribed from notch-1 locus. The miRNA downregulates cdk-18, a cyclin-dependent kinase that regulates M-G1 transition in cycling cells^2,3^. Suppression of cdk-18 triggers mitophagy and autophagy. Due to high autophagic flux, oestrogen receptor-1^+^/progesterone receptor^+^/p53^+^ (Esr1^+^/Pr^+^/p53^+^) SKBR3 cells are coerced into an Esr1^−^/Pr^low^/p53^−^profile. Increased mitophagy in combination with proteasomal degradation of p53 transiently arrests the cycling cells at G0 and enhances radio-resistance of the SKBR3 population. These findings highlight the impact on cancer therapy of non-encoded neoplastic resistance, arising as a consequence of miRNA-mediated autophagic reprogramming that uncouples phenotype and genotype.

## Introduction

Tumour resistance attributed to clonal evolution of neoplastic cells, poses a major challenge for cancer therapy^4^. Clonality of neoplastic cells is mainly considered to reflect heterogeneity of mutational landscape^5^. In the proposed linear evolutionary model, the mutational landscape is shaped by selection for phenotypes that improve the fitness profile of neoplastic cells in a step-wise and protracted manner. Recent reports suggest, however, alternative routes to tumour resistance. One such mechanism is clonal competition that leads to oscillatory dominance of resistance subclones in response to treatment^6^. Similar reports of alternating phenotypic reversal^6^ suggest a more dynamic non-linear nature of tumour resistance that is not necessarily encoded. While, upstream mediators of “non-encoded” tumour resistance remain largely unknown, improved survival of a neoplastic population is underpinned by enhanced apoptotic threshold^7,8^.

Enhanced apoptotic threshold is typically caused by mutations that impair genomic surveillance mechanisms. In particular, loss-of-function mutations of the tumour suppressor gene p53 are associated with resistance to apoptosis^9^. While p53 has a short half-life in normal cells, DNA damage, among other sources of cellular stress, can stabilise p53^10^. The stabilised p53 in turn communicates cell cycle arrest and activates apoptotic signalling^10^. However, apoptosis is avoided if p53 activates concomitant autophagy as a pro-survival mechanism^11^. The increased autophagic flux promotes rapid degradation of p53 and prevents excessive accumulation of stable p53 and subsequent apoptosis^11^. Neoplastic cells commonly utilise the latter phenomenon to bypass p53-induced apoptosis via increased autophagic flux^12^. To that end, microRNAs are emerging as key regulators of autophagy in neoplastic cells^13^. While microRNAs inhibit various stages of the autophagy cascade from induction to vesicle nucleation^14^, there is no evidence for the positive induction of autophagy. Herein, we report efficient induction of autophagy by miRNA-4673 encoded in intron 4 of human notch-1 locus. The miRNA-induced autophagic flux depletes cytoplasmic p53 and improves post-radiation survival of neoplastic cells. The observed non-encoded induction of resistance is underpinned by miRNA-mediated cytoplasmic reprogramming.

## Results

**Fig. 1:**
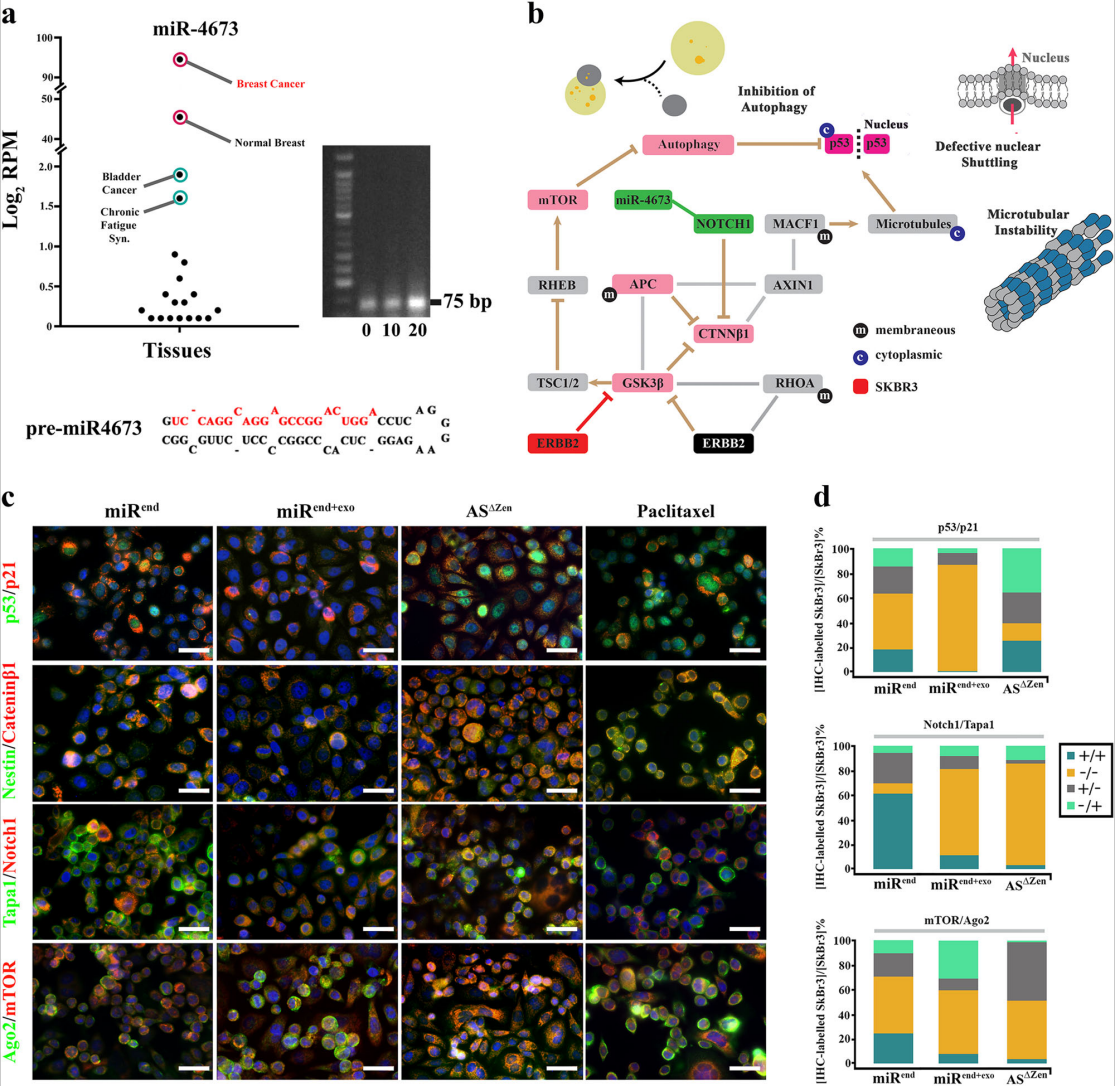
Uncoupling of genotype/phenotype propelled by miR4673. **a** Endogenous miR4673 expression in various human tissues extracted from miRmine database^78^. See methods for detailed list of tissue samples represented in dots. Gel shows endogenous expression of miR4673 in SKBR3 cells detected by stem-loop PCR method at sequential time points 10 min. apart. **b** Diagrammatic representation of genotype/phenotype interface in Erbb2^high^ SKBR3 cells. Inhibition of autophagy, microtubule destabilization, and nuclear import defects characterise the phenotype of SKBR3 cells. **c** Immunohistochemical panel highlighting the impact of miR4673 on bistable population-level transition of neoplastic cells. Application of the exogenous miRNA (miRend + exo) leads to repression of p53, cateninβ1 and Notch-1 and suppression of the endogenous miRNA (AS^∆Zen^) reverses the observed trend. The molecular fingerprint subsequent to amplification of miR4673 strongly contrasts with the molecular signature after application of Paclitaxel (Scale bars = 40 μm). **d** Quantification of immuno-labelling profile from **c** following up- and downregulation of the endogenous miRNA activity. Dynamics of four non-encoded clonal profiles are demonstrated by the presence or absence of the first and second markers in four combinations ( + / + , −/−, + /−, −/ + ) in stacked bar plots. The −/− to + / + conversion signifies non-encoded modulation of p53/p21/notch-1 profile by the miRNA

MiR4673 was initially detected in clinical samples of breast cancer patients^15^. Transcript level of miR4673 in neoplastic breast tissue is significantly higher than other tissues (Fig. 1a). We, therefore, selected the SKBR3 mammary carcinoma cell line with an extra copy of ligand-independent Erbb-2 (Her2) and high endogenous expression of miR4673 (Fig. 1a) to study potential involvement of miR4673 in induction/suppression of non-encoded resistance mechanisms.

### **Amplified miR4673 activity uncouples phenotype from genotype**

In SKBR3 cells, signalling input from an additional copy of erbb-2 informs the neoplastic phenotype. Hyperactive erbb2 inhibits glycogen synthase kinase-3β (GSK3β)^16^ that activates MDM2 by phosphorylation. As a result, MDM2-dependent degradation of p53 is abolished^17^ (Fig. 1b). Likewise, catenin-β1 (key Wnt mediator) evades GSK3β-mediated degradation (Fig. 1b). We initially investigated the impact of miR4673 signalling on p53 and catenin-β1 as phenotypic landmarks of SKBR3 cells. Amplification of endogenous miRNA signalling (miR^end^) by an exogenous dose of the naked pre-miRNA (miR^end+exo^) significantly expanded the p53^-^/p21^−^ population at the cost of p53^+^/p21^+^ cells within 24 h (45 to 86%; Fig. 1c, d). Quenching of the endogenous miRNA by application of an antisense RNA (AS^Δzen^, 100 nM, 2 × 10^5^ cells) largely reversed the observed phenomenon (45 to 14%; Fig. 1c, d) within the same timeframe. A similar bistable trend following amplification/inhibition of the endogenous miRNA signalling was noted for catenin-β1 (Fig. 1c). Concurrent reduction in miR^end+exo^ cells of both intracellular Notch-1 and antagonistic catenin-β1^18^, hinted at a non-selective depletion of the intracellular protein pool (Fig. 1c, d).

MiR4673 does not target p53, catenin-β1, notch-1 and erbb-2 directly. Hence, offsetting the anti-catabolic signalling input from hyperactive Erbb2 can be accomplished by post-transcriptional (Ago2-dependent RNA silencing) and/or post-translational (mTOR-dependent autophagy) mechanisms (Fig. 1c, d bottom). As predicted, miR^end+exo^ cells demonstrated Ago2^high^/mTOR^low^ profile in contrast to the Ago2^low^/mTOR^high^ fingerprint of AS^Δzen^ cells. The miR-mediated Ago2^high^ profile communicates enhanced Ago2-dependent RNA silencing^19^. The mTOR^low^ profile, on the other hand, is consistent with increased autophagic flux^20^. In addition to autophagy, downregulation of mTOR reduces protein synthesis via ribosomal S6 kinase^21^ and 4E-BP1 (Eukaryotic Translation Initiation Factor 4E Binding Protein 1)^22^. The findings suggest that the Ago2^high^/mTOR^low^ profile uncouples genotype and phenotype by non-selective depletion of intracellular protein reservoir. We, therefore, investigated potential impact of phenotypic uncoupling on the breast cancer diagnostic fingerprint^23^ informed by availability of Esr1, Pr, and Erbb2 proteins.

### Phenotype/genotype uncoupling distorts the diagnostic portrait of neoplastic cells

**Fig. 2:**
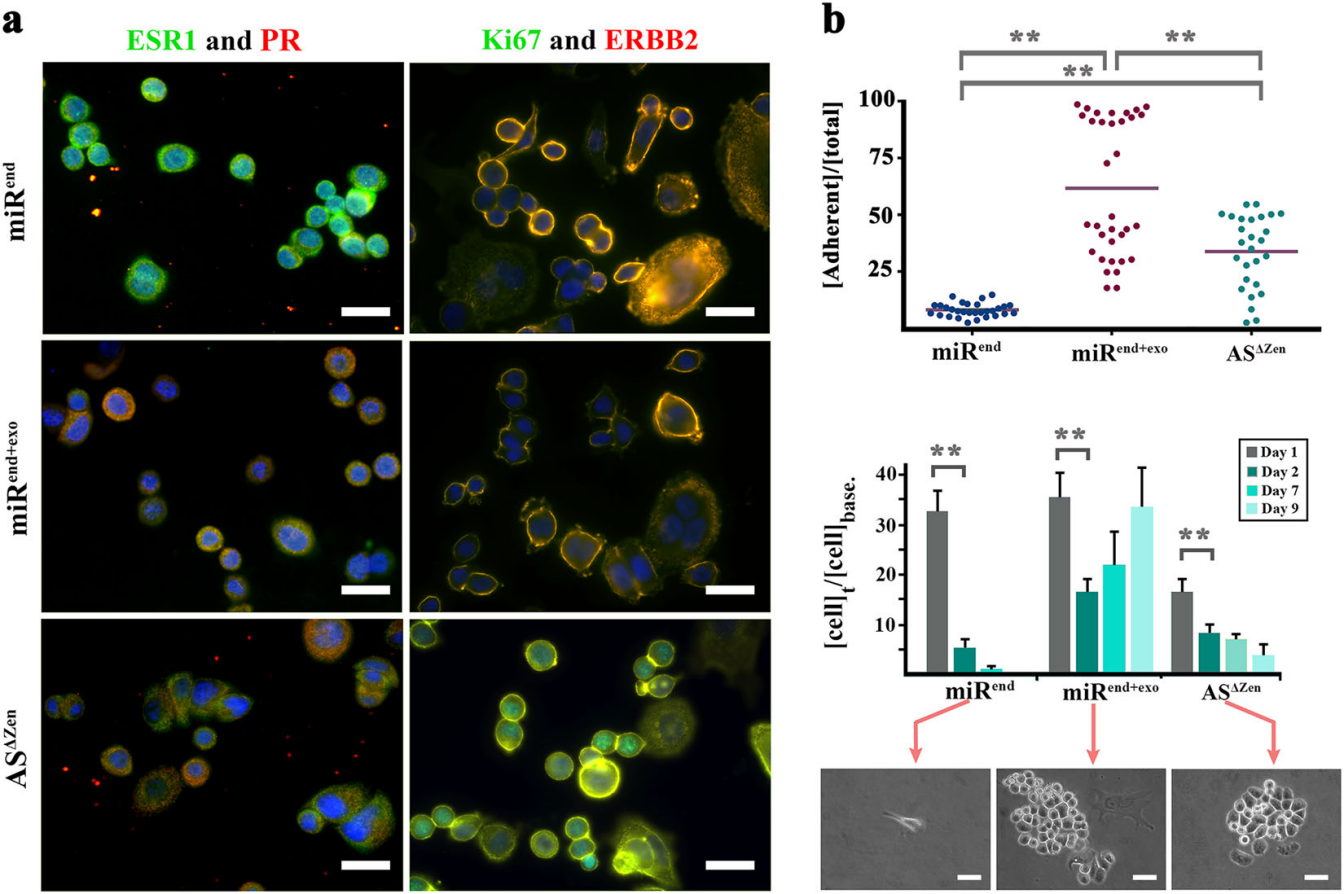
Phenotypic manifestations of nuclear uncoupling. **a** Regulation by miR4673 alters the diagnostic molecular profile of SKBR3 cells. Note the absence of Esr-1 and reduced expression of Pr and Erbb-2 subsequent to amplification of miR4673. **b** Graphs demonstrate adherence (top) and post-radiation survival (bottom) of neoplastic cells. Phase contrast images show viable adherent cells. Note the improved adherence and fast post-radiation recovery after amplification of the endogenous miRNA. ** indicates *p* < 0.01

Forced signalling by the microRNA (miR^end+exo^) coerced an Esr^−^ and Pr^low^ molecular profile in the neoplastic cells (Fig. 2a) similar to the miR-induced depletion of catenin-β1 and p53. The miRNA also restored normal membranous representation of Erbb2 in Erbb2^high^ neoplastic cells by overriding transcriptional input from the extra copy of the gene (Fig. 2a). In agreement with our findings, proteasomal degradation can effectively exhaust the cellular reservoir of Erbb2^24^ and ESR-1^25^ despite active transcription and translation. The supervisory dominance of proteasomal activity over transcription is often invoked during development to offset transcriptional noise and induce signalling robustness^26^. The same capacity is recapitulated in response to stressors where p27 induces autophagy and cell cycle arrest as a pro-survival mechanism^27^. Interestingly, we noted re-emergence of nuclear Ki-67 (Fig. 2a) following inhibition of the endogenous miRNA (AS^Δzen^) that suggests entry into the interphase^28^. One interpretation is that enhanced protein degradation in miR^end+exo^ SKBR3 cells impacts on availability of cyclins and arrests the cells at G0 phase. In a parallel scenario, serum starvation arrests cycling cells at G0 by upregulating autophagy^29^. The quiescent cells arrested at G0 are expected to become more resistant to stressors due to autophagy. As expected, the miR^end+exo^ cells changed morphology, became more adherent and demonstrated higher survival rate and faster recovery following UVC irradiation (Fig. 2b). Enforced adherence to matrix suggested stabilised integrin-based focal adhesions. Focal adhesion kinase signalling, in turn, inhibits cyclin-dependent kinase inhibitors p21 and p27^30^ and improves tolerance to DNA damage^31^ caused by irradiation. We, therefore, sought a molecular mechanism that explains the increased protein degradation and G0 arrest instructed by miR4673 signalling.

### A faux stress signal improves fitness of neoplastic cells by invoking a G0 molecular signature

**Fig. 3:**
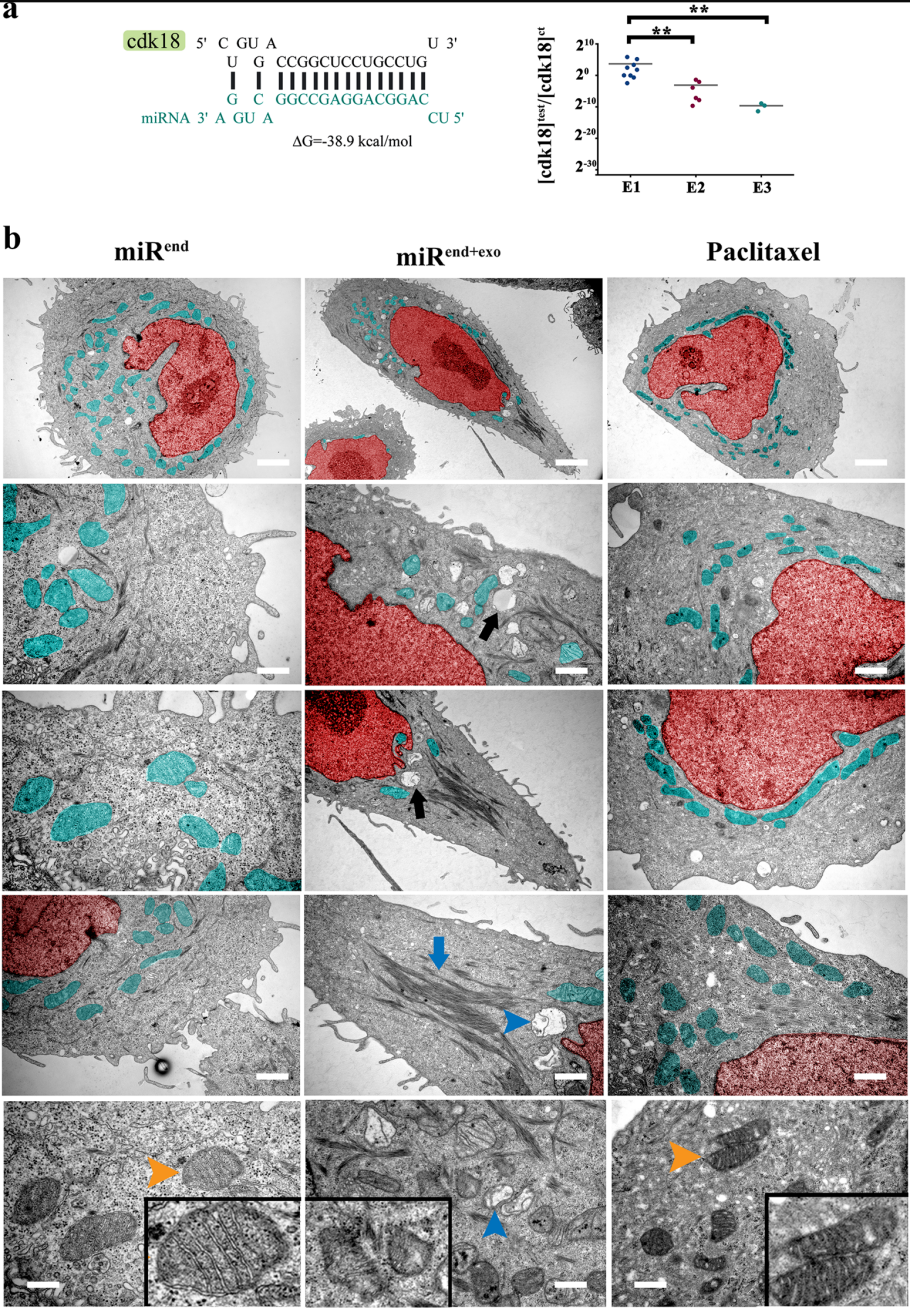
MiR4673 enhances autophagy and induces mitophagy. **a** MiR4673 can hybridise to cdk-18 with high affinity (left). Application of miR4673 inhibited cdk-18 in a dose-dependent manner. E1, E2, E3 are sequential electroporations 24 h apart (** indicates *p* < 0.01). **b** Ultrastructural changes of SKBR3 cells after amplification of the endogenous miRNA (Red: nucleus & Aqua: mitochondria). Note stabilised actin microfilaments (middle) and autophagy (black arrow) subsequent to amplification of the endogenous miRNA. The observed ultrastructural changes following amplification of miR4673 contrast sharply with the numerous active perinuclear mitochondria detected after application of Paclitaxel (right). Amplification of miR4673 signalling triggered mitophagy (bottom, blue arrowhead) in contrast to Paclitaxel-induced activation of mitochondria (bottom right, orange arrowhead) Insets show mitochondrial ultrastructure (scale bars: top = 2 μm, middle left = 0.5 μm, middle = 0.7 μm, middle right = 1 μm, bottom left, bottom left = 0.2 μm, bottom middle = 0.6 μm, bottom right = 0.3 μm, bottom left)

**Fig. 4:**
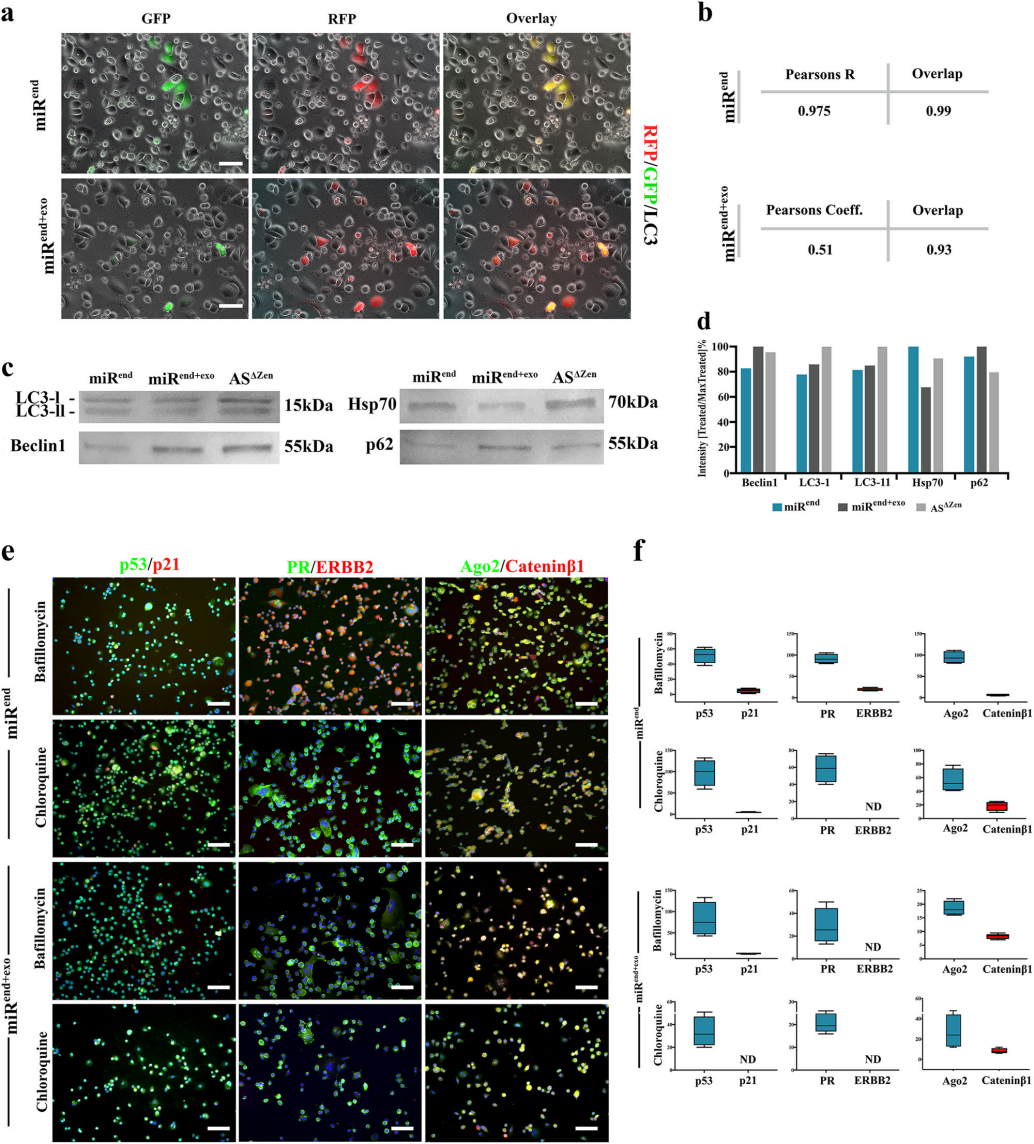
Enhanced autophagic flux underpins the altered molecular fingerprint of amplified miR4673 signalling in SKBR3 cells. **a** Live-imaging analysis of miR^end^ and miR^end+exo^ SKBR3 cells 24 h after transfection with RFP/GFP‐tagged LC3 reporter plasmid, ptfLC3 (Scale bars = 40 μm). While miR^end+exo^ SKBR3 cells express RFP, miR^end^ cells express both RFP and GFP. The latter is consistent with the enhanced incorporation into the autophagosomes and fusion with lysosomes of RFP/GFP-LC3 subsequent to the amplification of endogenous miR4673. **b** Co-localisation analysis of RFP and GFP (based on Spearman’s coefficient and overlap) confirmed uncoupled RFP and GFP signals in miR^end+exo^ cells as a result of upregulated autophagy. **c** Western blot analysis of autophagy-related proteins LC3, Beclin-1, p62 (SQSTM1), and Hsp-70. The reduced level of LC3-II in miR^end+exo^ SKBR3 cells due to autophagy was consistent with live-imaging analysis of ptfLC3-transfected cells. Likewise, enhanced levels of Beclin-1 and p62 (SQSTM1) proteins in miR^end+exo^ SKBR3 cells indicated amplified induction/assembly phase of autophagy. Eventually, we investigated the availability of HSP-70 as a chaperone for protein folding and autophagy. HSP-70 was retrieved at a lower level from miR^end+exo^ SKBR3 cells consistent with enhanced degradation phase of autophagy. **d** Semi-quantitative analysis of **c** based on intensity of the bands normalised to the maximum intensity (100%). **e** Reduction of autophagic flux by the application of Bafilomycin A1/chloroquine restored the reduced cytoplasmic pool of p53, PR, and Cateninβ1 in miR^end+exo^ SKBR3 cells to a level comparable to that of control miRend cells (Scale bars = 100 μm). **f** Quantification of immuno-labelling profile from **e** following the reduction of autophagic flux by the application of Bafilomycin A1/chloroquine

We identified Cdk-18, the closest mammalian homologue of yeast Pho85 cyclin-dependent kinase, as a potential target of miR4673 (Fig. 3a). In yeast, Pho85 acts as a nutrient sensor^32^ that licences M-G1 transition in cell cycle^3^. Pho85 downregulates autophagy^33^ to propel cell cycle progression after mitosis. In response to nutrient deprivation, pho85 induces arrest at G0 to improve the survival of yeast cells. Likewise, the human homologue (PCTAIRE Protein Kinase 3 or cdk-18) acts downstream of cyclin-A2^34^ to drive cell cycle progression. The impact on cell cycle of Cdk-18 occurs in part by relieving endoplasmic reticulum stress^35^ and concomitant autophagy^36^ to restore activity of cyclins that drive progression through G1/S^37^. Sequential application of exogenous miRNA (200 nM, 2 × 10^5^ cells) resulted in near-complete post-transcriptional inhibition of cdk-18 (Fig. 3a). The inhibition of cdk-18 inactivates cofilin^34^ that manifests as reduced actin depolymerisation in miR^end+exo^ cells (Fig. 3b). The miR4673-mediated suppression of Cdk-18 also explains the upregulated autophagy/mitophagy in miR^end+exo^ cells^35^ (Fig. 3b). The diminished mitochondrial activity subsequent to mitophagy not only contributes to radio-resistance^38^ (Fig. 2b) but can also indirectly trigger redox-dependent polymerisation of actin microfilaments^39^ (Fig. 3b). Enhanced mitophagy downstream to miR4673 signalling effectively offsets Paclitaxel-induced perinuclear localisation of mitochondria shown to trigger apoptosis by imposing an oxidative burden on the nucleus^40^ (Fig. 3b). Arguably, communication of faux nutrient deprivation signal (Cdk-18^−^ equivalent to Pho85^−^) by miR4673 triggers a key stress-response with concomitant autophagy/mitophagy that improves plasticity and survival capacity of neoplastic cells. The application of exogenous miRNA-4673 enhanced the induction of autophagy and progression to autophagosome formation and subsequent degradation of the cargo (Fig. 4a–d). The inhibition of autophagic flux partially restored the molecular profile of SKBR3 cells in miR^end + exo^ cells (Fig. 4e, f). The latter also explains entry into interphase following the suppression of miR4673 (ki-67^+^ AS^Δzen^ cells in Fig. 2a) that communicates an absence of stressors. We further dissected the impact of faux metabolic stress (cdk-18^−^ profile) on transcriptional fingerprint of cycling miR^end+exo^ cells.

**Fig. 5:**
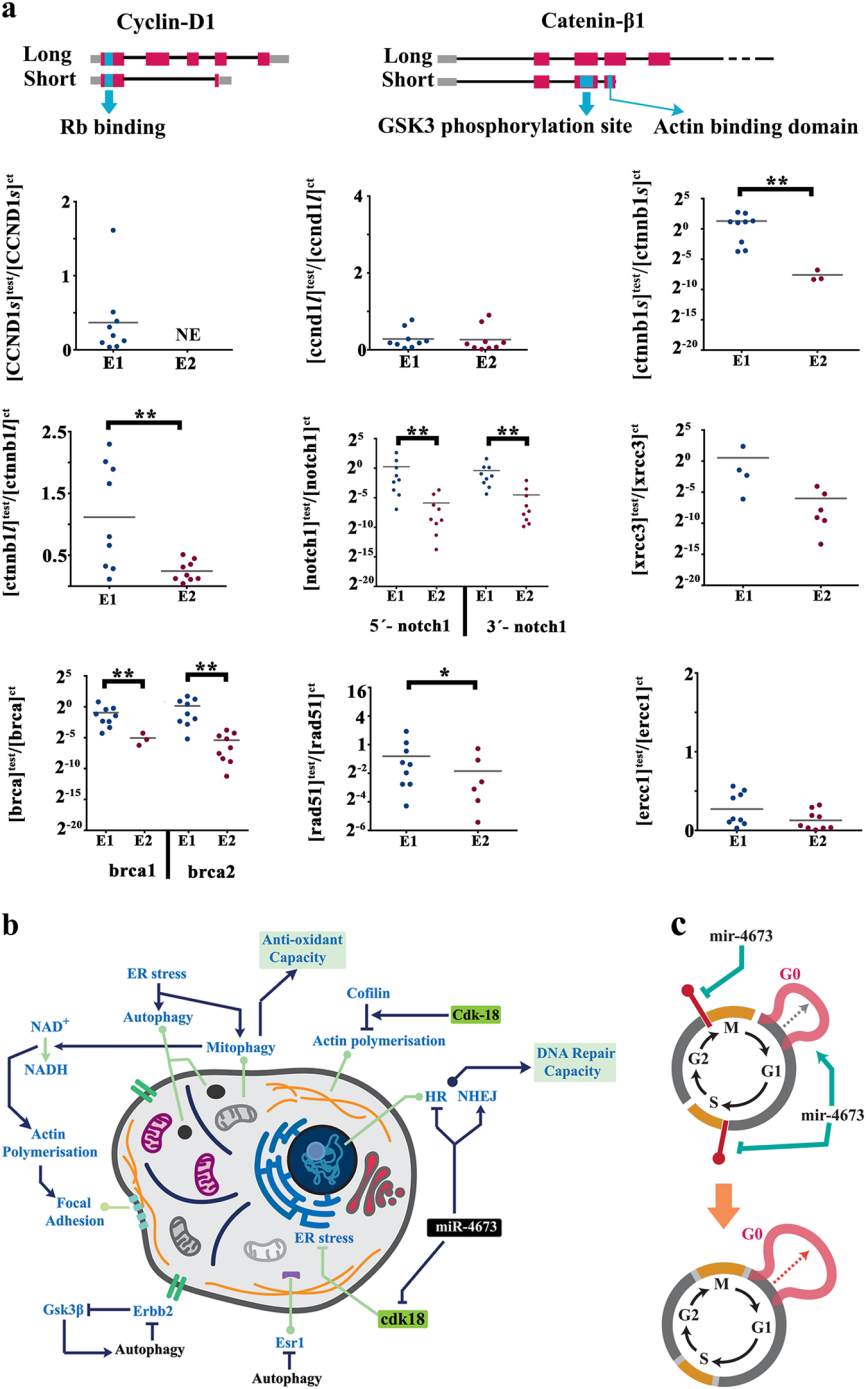
Amplified miR4673 signalling coerces transcriptional fingerprint of SKBR3 cells into a G0 signature. **a** The short isoform of cyclin-D1 only encodes for retinoblastoma binding domain. The short isoform of catenin-β1 only encodes for the phosphorylation domain of the protein. The graphs show altered molecular fingerprint of SKBR3 cells following the amplification of endogenous miR4673 signalling (* indicates *p* < 0.05, ** indicates *p* < 0.01). E1 and E2 are sequential electroporations 24 h apart. We used two separate sets of primers to fingerprint the 5ˊ and 3ˊ notch-1 locus. **b** Schematic demonstration of system-level miR4673 interactions that improve fitness profile of neoplastic cells. Interactions triggered by the miRNA signalling eventually improve anti-oxidant defence capacity and accelerate DNA repair mechanisms through non-homologous end-joining. **c** Schematic representation of cell cycle shows prolonged G0 subsequent to the amplification of the endogenous miRNA activity that instructs inhibition of G1/S and G2/M cycle checkpoints simultaneously

The cateninβ1-cylinD1-retinoblastoma axis, as the major propellant of cell cycle^41,42^, was focused upon. A key transcriptional signature of miR^end+exo^ cells was downregulation of cyclin-D1 short isoform that lacks the cyclin box required for cdk4/6-mediated phosphorylation of retinoblastoma protein^41^ (Fig. 5a). A similar trend was evident in alternative splicing of catenin-β1 following the amplification of miR4673. The transcriptional suppression of truncated cyclin-D1 eliminates decoy protein (alternatively spliced with no cyclin box) that can inhibit the Rb-E2f cascade at G0 (Fig. 5a). The elimination of decoy truncated catenin-β1 (with actin binding and phosphorylation domains) enhances the phosphorylation likelihood of full length protein required for *trans*-signalling^43^ (Fig. 5a). Transcriptional repression of Notch-1 (antagonist of catenin-β1) further energises the activated cateninβ1-cylinD1-Rb axis at M-G1 transition during cell cycle. Activation of the latter axis in G0-arrested cdk-18^−^/miR^end+exo^ cells occurs in anticipation of progress into G1. Enhanced autophagy, by proteasomal degradation (Fig. 1c), offsets the activity of cateninβ1^−^cylinD1-Rb axis and arrests the miR^end+exo^ cells at G0. Another prominent transcriptional change following amplified miR4673 signalling was silencing of Brca2 and X-Ray Repair Cross Complementing 3 (Xrcc3). Brca2 and Xrcc3 are critical components of homologous recombination (HR) machinery^44,45^. Inhibition of BRCA2 and XRCC3, which suggests inactivation of HR, is also a signature of G0/early G1^46^. The inhibition of HR alters the balance in favour of error-prone non-homologous end-joining (NHEJ). The remarkably accelerated dynamics of NHEJ compared to HR in repairing DNA breaks (≈30 min versus > 7 h)^47^ contributes to post-radiation survival and recovery^48,49^ of miR^end+exo^ SKBR3 cells. Transcriptional silencing of Brca2 may in part reflect the depletion of upstream activator ESR-1 by autophagy (Fig. 2a)^50^. On the other hand, the mitophagy-mediated switch to glycolysis exhausts the cellular supply of NAD^+^ and decreases NAD^+^/NADH ratio that in turn triggers transcriptional silencing of brca-1^51^. The findings point to a stress signature invoked by endogenous miR4673 signalling compatible with G0 molecular fingerprint (Fig. 5b). The latter is aligned to the ancestral role of Pho85 (Cdk-18 homologue) in signalling G0 arrest in response to stressors^3^. Hence, suppression of cdk-18 by miR4673 communicates a faux stress signal analogous to stress-mediated inhibition of Pho85 that triggers pro-survival autophagy and stress adaptation. In addition to G0 lengthening, downregulation of brca1, brca2, and xrcc3 combined with proteasomal degradation of p53 can potentially relax the G1-S checkpoint and accelerate interphase during which cells are more susceptible to DNA damage (Fig. 5c). Acceleration of interphase by suppressing the G1 checkpoint is a feature of embryonic stem cells^52^ and it is not uncommon for neoplastic cells to recapitulate aspects of ontogeny. In our proposed model for reprogramming of cell cycle by miR4673 activity, miR^end+exo^ cells dwell longer in G0 (stress-resistant phase) and progress more rapidly through G1 (sensitive phase).

**Fig. 6:**
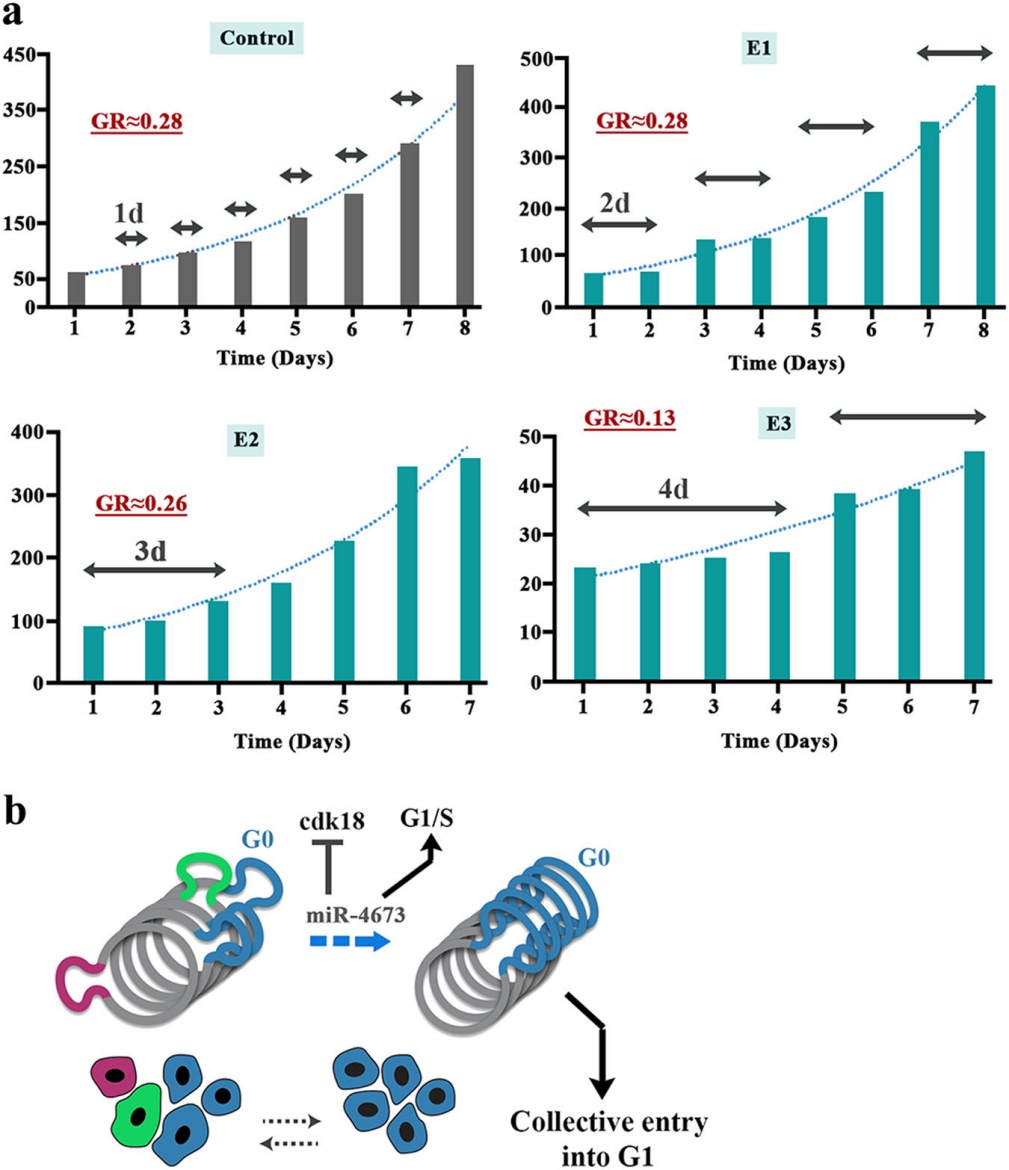
SKBR3 population growth dynamics adapts to reprogramming of cell cycle by amplified miR4673. **a** E1 to E3 refer to boosted applications of the exogenous miRNA every 24 h. The horizontal arrows demonstrate periodicity of dormancy in each group (GR: exponential growth rate). **b** The schematic model explains non-homogeneous growth pattern of part a based on the activity of miR4673. The miRNA transient synchronises the SKBR3 cell at G0 by inhibiting cdk-18 and subsequently accelerates G1-S transition by autophagy-mediated depletion of p53

We confirmed miR4673-induced cell cycle reprogramming by dissecting the population growth dynamics of miR^end+exo^ cells SKBR3 cells. In contrast to smooth exponential growth of control cells, sequential application of the exogenous miRNA induced periods of dormancy followed by episodic population growth (Fig. 6a). This behaviour was consistent with transient cell cycle arrest at G0 (dormancy) followed by fast synchronised cycling and division (episodic population growth) (Fig. 6b). The unchanged growth rate despite arrest at G0 indicated a reciprocal shortening of interphase (Fig. 6a). Reprogramming of cell cycle by miR4673 can potentially improve resistance profile of the neoplastic cells by lengthening G0 (resistant phase) and simultaneous shortening of interphase (sensitive phase). We next explored endogenous transcriptional oscillations of miR4673 and the associated downstream genes that instruct windows of resistance in neoplastic populations. The cells were first synchronised at G0 by serum starvation. Subsequent to addition of serum, a high-resolution temporal fingerprint of endogenous miR4673 and associated genes, that instruct the population fitness of SKBR3 cells, was generated.

### Endogenous oscillations of miR4673 and the downstream cascade portray a variable resistance landscape

**Fig. 7:**
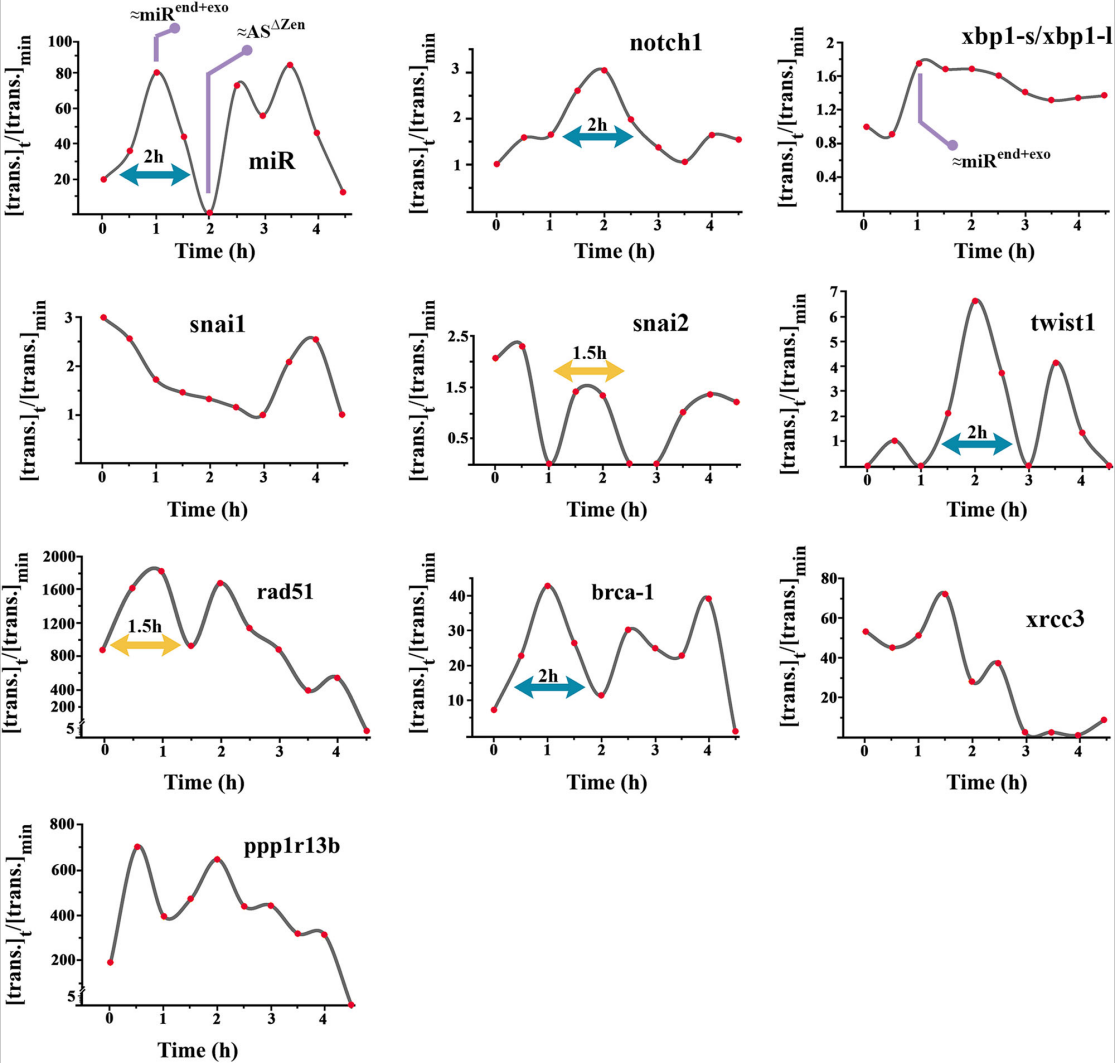
Endogenous transcriptional oscillations of miR4673 are coupled to oscillations of resistance-inducing genes. Temporal fingerprint of miR4673 and associated responsive genes synchronised by serum starvation. Note the 1.5–2-hour periodicity of expression. The *y*-axis shows expression level of genes at time point t ([trans.]_t_) normalised to the minimum expression value ([trans.]_min_). β-actin was used as the reference gene

Transcriptional activity of the endogenous miRNA and the host gene notch-1 oscillated with a periodicity of 2 h (Fig. 7). These results are consistent with the reported periodicity of notch-1 oscillations^53^. Notably, amplitude of the miRNA oscillation ([max]:[min]≈80-fold) was much higher than notch-1 oscillation ([max]:[min]≈3-fold). In addition, notch-1 oscillation lagged behind that of the endogenous miRNA by 1 h. Concurrent oscillation of the endoplasmic reticulum stress was measured based on shift in the alternative spliced product of X-Box Binding Protein 1 (xbp-1)^54^. High endoplasmic reticulum stress (characterised by abundance of short alternative splicing product of xbp-1) temporally accorded with peak miR4673 transcriptional activity (Fig. 7a). The transcription of rad51 recombinase (a core component of HR) and PPP1R13B (ASPP1; a positive regulator of p53 activity) and xrcc3 showed a periodicity of 1–1.5 h and was repressed concurrent with the second peak activity of miR4673 (Fig. 7). Transcription from brca-1 was, however, totally in phase with oscillations of miR4673 and showed a periodicity of 2 h. We fingerprinted Snail family members as potent inducers of G0 arrest and neoplastic resistance^55^. The transcriptional fingerprint of twist-1, a *trans*-acting partner for notch-1^56^, was temporally aligned to the latter gene and showed a periodicity of 2 h (Fig. 7). The transcriptional oscillation of snai-2 as a downstream target of Notch-1^57^ accorded with twist-1 expression (periodicity: 1.5 h). These oscillatory fingerprints foreshadow the alignment of endogenous miR4673 oscillations to the windows of resistance that occur at intervals of ≈2 h. We, therefore, tested the contribution of miR4673 to the adaptation of SKBR3 cells to the acute stressor, ionising radiation.

### Endogenous signalling by miR4673 improves the survival likelihood of SKBR3 cells

We modelled the contribution of endogenous miR4673 to post-radiation survival of the neoplastic cells based on Poisson statistics and assumption of “single-target single-hit”^58^. The binary model assumes that a single exposure to the radiation is sensitive enough to kill the impacted cell. Hence if the radiation hits a cell “*k*” times, the likelihood of escape from the impact of radiation or P(*k* = 0) in Poisson probability space corresponds to:

$$P\left( k \right) = \frac{{m^k}}{{k!}}e^{ - m}$$

$$P\left( {k = 0} \right) = \frac{{m^0 \times e^{ - m}}}{{0!}} = e^{ - m}$$

**Fig. 8:**
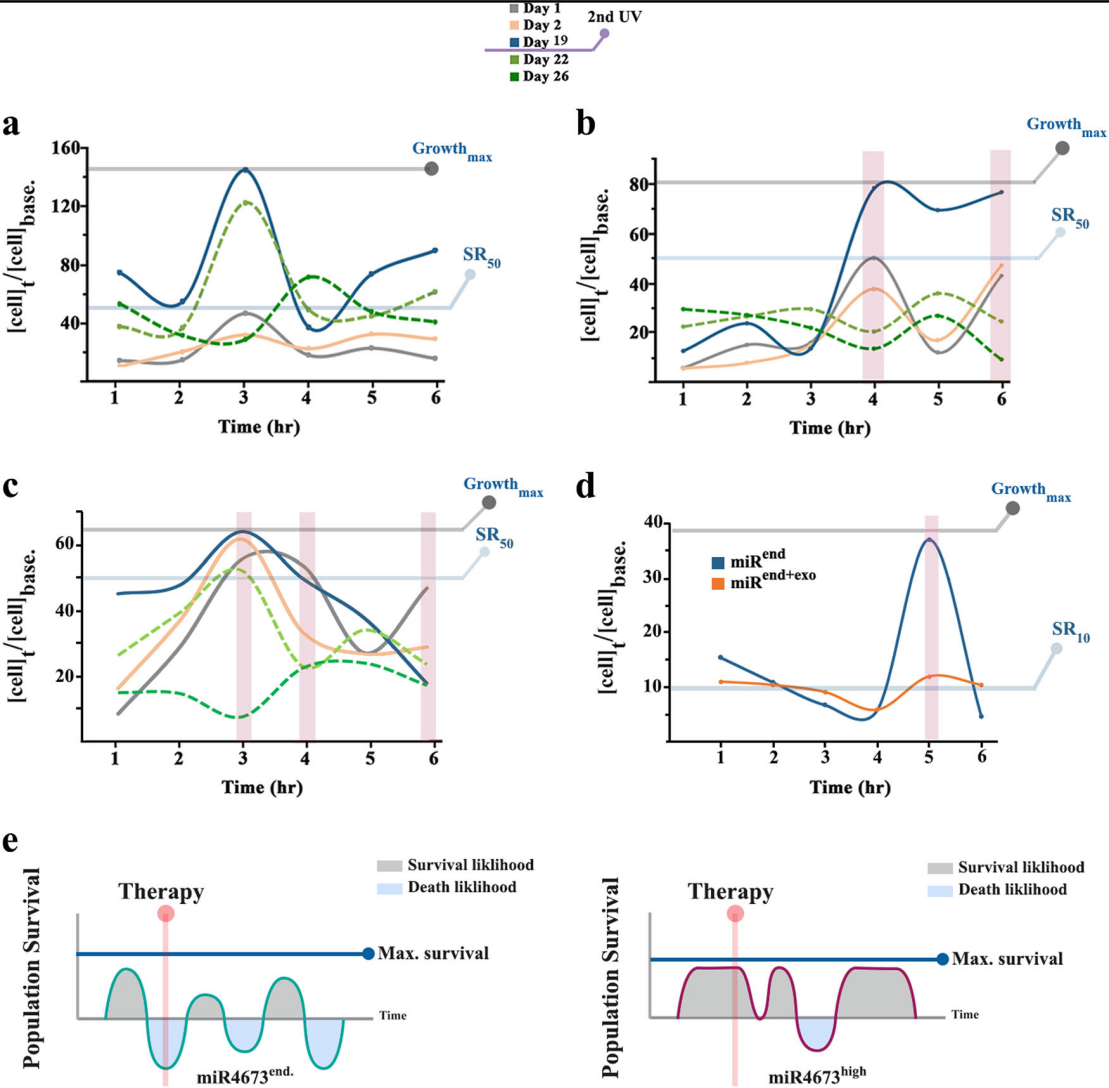
MiR4673 improves the survival rate of SKBR3 cells. **a** Post-radiation survival of control SKBR3 cells. **b** Post-radiation survival of miR^end+exo^ SKBR3 cells (100 nM exogenous miR4673). **c** Post-radiation survival of miR^end+exo^ SKBR3 cells (200 nM exogenous miR4673). The surviving cells were exposed to a second dose of radiation after day 19. Note the reversal of survival rate at *t*_3_ of control cells and *t*_3_, *t*_4_, *t*_6_ of miR^end+exo^ cells (dotted lines). **d** Post-radiation survival (day 1) of control miR^end^ and miR^end+exo^ SKBR3 cells (200 nM exogenous miR4673) subsequent to inhibition of autophagy by Chloroquine. Note the reduced survival rate in miR^end+exo^ similar to that observed for miRend cells. **e** Schematic diagram demonstrates the contribution of miR4673 to the fitness landscape of SKBR3 cells. The amplified activity of miR4673 increases the incidence of resistant periods. The higher incidence of resistant temporal windows (bottom graph) increase the likelihood of treatment in a period where neoplastic cells are refractory

Where m (dose correction parameter) is proportional to the probability of exposure to the radiation. To measure endogenous variability in resistance, SKBR3 cells were exposed to ionising radiation at sequential time points 1 h apart. Notably, the post-radiation survival rate (SR) of control SKBR3 cells (with endogenous miR4673) oscillated with a periodicity of 2–3 h (Fig. 8a). The endogenous difference between the maximum SR (*t*_3_) and the minimum SR (*t*_1_) in the control group based on Poisson statistics

$$P(k = 0) = e^{ - m} \Rightarrow {\mathrm{ }}m_{}^{t_1}/m^{t_3} = Ln(SR^{t_1})/Ln(SR^{t_3}) \\ = Ln(0.15)/Ln(0.47) = 2.5$$

indicated ≈2.5-fold reduction of the impact of radiation on population from *t*_1_ to *t*_3_ (measured based on day 1 post-radiation SR). We then translated the ≈2.5-fold reduction of radiation impact at a population-level to the improved fitness of individual cells based on the growth of parameter “m” from *t*_1_ to *t*_3_

$$P(k = 0) = e^{ - m} \Rightarrow m_{}^{t_3} - m^{t_1} \\ = Ln(0.47) - Ln(0.15) = 1.14$$

The growth by 1.14 unit of the parameter “*m*”, that is given a default value of 1 based on the “one hit one target” model, corresponds to a halved probability of death by irradiation for individual cells. Therefore, two hits are required to cause lethality based on Poisson’s model. On the other hand, a single exogenous dose of miR4673 (Fig. 8b, c) reduced the impact of radiation on neoplastic cells at *t*_4_ by

$$m_2^{t_4}/m^{t_4} = Ln(SR_2^{t_4})/Ln(SR^{t4}) \\ = Ln(0.18)/Ln(0.49) = 2.4$$

where m_2_ and m refer to irradiated miR^endo+exo^ and control groups, respectively. Enhanced survival rate after exogenous application of miR4673 at *t*_4_ (2.4-fold) approached the maximal endogenous radio-resistance (2.5-fold). Further, amplified miR4673 signalling enhanced the incidence of radio-resistant periods by two fold at 100 nM (Fig. 8b) and by three fold at 200 nM (Fig. 8c). Remarkably, a second dose of radiation at day 19 triggered a reversal of the survival profile at *t*_3_ in the control group (dotted lines in Fig. 8a–c) and at *t*_4_ in the miR^endo+exo^ population consistent with the non-encoded nature of radio-resistance. The application of Chloroquine reduced the mean survival rate in both miR^end^ and miR^end+exo^ SKBR3 cell to ≈10% (Fig. 8d). We, therefore, propose a model for the miR473-induced enhancement of neoplastic fitness profile (Fig. 8e) where 50% increased resistance of individual neoplastic cells anticipates a 2.5-fold improvement of the population survival rate at any time point. In our proposed model, miR4673 signalling also increases the incidence of resistant temporal windows.

## Discussion

The findings reported herein attest to plasticity of molecular signatures that inform non-encoded resistance in a neoplastic population. Plasticity results from miR4673 signalling that upregulates proteasomal degradation and overrides the transcriptional fingerprint of SKBR3 cells. The resultant phenotypic uncoupling significantly improves fitness profile of the neoplastic population. Phenotypic uncoupling is particularly manifest in the repressed profile of p53 following the amplification of miR4673.

Tumour suppressor p53 is a key target of human oncogenic miRNAs. MiR-125b, for example, downregulates p53 by targeting the 3ˊ-untranslated region (UTR) of the transcript in human neuroblastoma cells and human lung fibroblast cells^59^. Likewise, miR504-mediated suppression of p53 enhances the resistance of neoplastic cells by increasing the threshold of apoptosis^60^. Hybridisation to the UTR, however, triggers partial inhibition of the targeted transcript^61^. In contrast, indirect inhibition of p53 by miR4673-mediated autophagy leads to near-complete suppression of the protein. Direct targeting of xrcc3 by miR4673 complemented indirect suppression of p53. Notably, loss-of-function mutations of XRCC3 attenuate the apoptotic signalling in neoplastic cells^62^. At a population-level, reprogramming of cell cycle by miR4673 (transient arrest at G0 followed by accelerated interphase) maximises the resistance of neoplastic cells. Enhanced autophagy is the key to the major phenotypic manifestations of miR4673 signalling.

The autophagy-mediated nuclear uncoupling reflects a developmental capacity to reprogram the proliferating cells by depletion of cytoplasmic protein repertoire^63^. The most remarkable example of the nuclear uncoupling occurs during reprogramming of the highly differentiated oocyte into undifferentiated zygote by complete degradation of maternal proteins^64^. Mitophagy is also commonly employed in physiological processes ^65^. Differentiation of the reticulocytes, for example, is governed by amplified mitophagy^66^. Recapitulation of autophagy-mediated reprogramming enables neoplastic cells to adapt to stressful situations ^67^. In response to the application of anticancer drugs autophagy can act as a pro-survival mechanism^68^. Autophagy also improves resistance to ionising radiation^69^. The induction of autophagy by miR4673 is unique as most reported human microRNAs are negative regulators of autophagy^14^. Some other aspects of the autophagic reprograming by miR4673 are also worthy of attention.

A distinguishing feature of signalling by miR4673 is system-level interactions that complement autophagy-mediated reprogramming and enhance the potency of the microRNA signalling. Suppression by autophagy of p53, for example, is complemented by transcriptional inhibition of xrcc3. Another distinguishing feature of miR4673 signalling is acute endogenous oscillations of the miRNA (every 2 h). These transcriptional oscillations are aligned to temporal behaviour of notch-1, the host locus of miR4673. Transcription from Notch-1 locus and the associated downstream mediators oscillates with a periodicity of 2 h^53^. The transcriptional oscillations of miR4673 accommodate windows of neoplastic resistance with potential implication for therapy. The phenotypic variability may also impact on the accuracy of diagnosis by distorting the molecular fingerprint of tumours. Finally, the non-encoded nature of the described reprogramming mechanism suggests that the capacity may be available to other neoplastic cells with active transcription from notch-1 locus. Even in the absence of active transcription, epigenetic remodelling may unlock this capacity and alter the behaviour and molecular profile of the neoplasm acutely.

From a diagnostic perspective, cytoplasmic reprogramming by autophagy can potentially alter the molecular fingerprint of neoplastic cells. Non-encoded reprogramming of SKBR3 cells by miR4673, for example, communicates a Esr1^-^/Pr^low^/erbb2^low^ subtype that can potentially indicate more aggressive treatment options. In parallel, the enhanced fitness profile not only improves real-time survival of neoplastic cells but also facilitates accumulation of iatrogenic genetic changes that may in turn propel a switch from non-encoded to encoded (genetic) resistance associated with poor prognosis^70^.

From a therapeutic perspective, non-encoded homogeneity is not without consequences. Our findings demonstrate that miR4673 improves radiation resistance of SKBR3 cells by 50% that translates into a 2.4-fold higher survival rate of the population. The improved fitness results from increased mitophagy that enhances anti-oxidant capacity of the neoplastic cells. The inhibition of homologous recombination machinery to empower non-homologous end-joining (NHEJ) cascade also improves DNA repair capacity. This effect is particularly manifest in resistance to ionising radiation where accumulating evidence indicates an indispensable role of NHEJ in repair of DNA damage^48,49,71^.

In conclusion, we have uncovered a non-coded route to tumour resistance (as opposed to mutational resistance) that results from intrinsic oscillations of miR4673 expressed at high levels in breast cancer. The signalling cascade utilised by the microRNA may be targeted to reveal the true molecular fingerprint of tumour by inhibition of the autophagic reprogramming. Suppression of the endogenous miRNA may also improve the response to therapy.

## Methods

### Materials and reagents

All chemicals were purchased from Sigma-Aldrich Inc. unless stated otherwise. All primers were purchased from IDT DNA.

### **Cell culture**

SKBR3 cell line was purchased from ATCC cell bank (ATCC^®^ HTB-30™). Cells were cultured in standard McCoys-5A medium (Sigma-Aldrich^®^, M4892) supplemented with 10% FBS (Gibco™) and were split at ≈75% confluence. All the experiments were performed at a similar confluency rate. Serum starvation method was utilised to synchronise the cycling cells at G0.

### Electroporation

For electroporation, cells were harvested and resuspended in a modified “Intracellular buffer”^72^ composed of HEPES (200 nM), MgCl_2_ (1.35 μΜ), Glutathione (10 μΜ) pH: 7.4. ECM-830 square wave generator (Harvard Apparatus BTX) was used for electroporation. The settings were 4 pulses of 1.6 kV/cm, 710 μs, at intervals of 1 s. After electroporation cells were transferred into T25 flasks (Falcon^®^). Naked microRNA-4673 was applied at 400 nM/2 × 10^6^ cells. Inhibition of the endogenous miRNA was achieved by utilising 2′-O-Methyl antisense RNA inhibitors (application dose: 200 nM/2 × 10^6^ HNPs) rendered RNAse-resistant by terminal N,N-diethyl-4-(4-nitronaphthalen-1-ylazo)-phenylamine (ZEN^TM^, IDTDNA).

### UVC irradiation

SkBr3 cells were were collected and transferred into a 6-well-plate and cultured for 2 days. A 30 Watt UVC generator at a distance of 40 cm from the plate was used as a source of ionising radiation. The UVC lamp was turned on for 3 × 20 s with 10 s intervals. The cells were then immediately supplemented with fresh McCoys media.

### RNA isolation

RNA was isolated using a Trizol reagent. Bromochloro (0.1 M) was added per 1 ml TRIzol^®^ Reagent (Ambion, 15596018). The tubes were vortex then centrifuged for 15 min at 12000 g at 4 ^°^C. The aqueous phase was mixed with 2 μl of 5 μg/μl linear polyacrylamide (ambion) and 500 μl of isopropanol. The solution was then vortexed then left to incubate at room temperature. The solution was then centrifuged for 1 h at 20,000 × *g* at 4^ °^C. The supernatant was discarded followed by the pellet being washed with 1 ml of ice cold 200-proof ethanol. The sample was then vortexed followed by centrifugation for 30 min at 12,000 × *g* at 4 ^°^C and the supernatant was discarded. The RNA pellet was left to air dry, then resuspended in 50 μl of RNAse free H_2_O (Ambion™ AM9937) and stored at ‒80 ^°^C.

### Reverse transcription

After DNase treatment, reverse transcription of extracted RNA was carried out by using a mixture of reverse primers (2pmole/primer, 4 μl total RNA, 1 μl dNTP mix (10 mM each), 4 μl of 5x First-Strand Buffer, 1 μl of 0.1 M diothiothreitol (DTT), 1 μl of RNAseOUT (40U/μl), 1 μl (200U) of Superscript-III reverse transcriptase. Reverse transcription was performed at 50^ °^C for 1 h.

### Real-time qPCR

Real-time quantitative PCR (38 cycles) was performed using SensiFAST™ SYBR^®^ Lo-ROX reagents (BIOLINE^®^). Reaction mix comprised of 2 μl of cDNA, 400 nM inner primers (1.5 μl/primer), 10 μl of 2x SensiFAST SYBR Lo-ROX Mix, and 5 μl of PCR-grade water on a Stratagene^®^ Mx3000P real-time PCR instrument. Average efficiency of PCR amplification for each gene of interest was quantified based on a linear regression model using the LineRegPCR software^73^. The relative (normalised to β-actin) expression ratio of gene of interest (test: control) was then calculated using the efficiency of amplification (Eff.) values based on the method proposed by Pffai^74^ as follows:

$${\mathrm{Ratio}} = \frac{{({\mathrm{Eff}}_{{\mathrm{tar}}})^{\Delta {\mathrm{ct}}\_{\mathrm{target}}({\mathrm{control}} - {\mathrm{test}})}}}{{({\mathrm{Eff}}_{{\mathrm{ref}}})^{\Delta {\mathrm{ct}}\_{\mathrm{reference}}({\mathrm{control}} - {\mathrm{test}})}}}$$

To generate the temporal fingerprints of genes, we used a variation of the above method:

$${\mathrm{Ratio}} = \frac{{({\mathrm{Eff}}_{{\mathrm{tar}}})^{\Delta {\mathrm{ct}}\_{\mathrm{target}}({\mathrm{control}} - {\mathrm{min}} )}}}{{({\mathrm{Eff}}_{{\mathrm{ref}}})^{\Delta {\mathrm{ct}}\_{\mathrm{reference}}({\mathrm{control}} - \min )}}}$$

Where min. refers to the time point with minimum expression level of the gene of interest and all other time points are normalised to min.

Melt curve analysis and visual inspection of the amplified products on 2% agarose gel electrophoresis confirmed the presence of target amplicons. Relative expression of transcripts for each gene was plotted as ratio between the average concentration of transcript in the test and control groups normalised to the average concentration of β-actin transcripts in both groups (above formula). The primers used in the current study are presented at Supplementary Table 1.

### Stem-loop PCR for detection of endogenous miRNA

Small RNA from proliferating HNPs was isolated using mirVana™ miRNA isolation Kit (Ambion) according to the manufacturer’s protocol. Detection of the miRNA-4673 was accomplished using stem-loop PCR as described elsewhere^75^. Specific primers were designed for reverse transcription and stem-loop RT-PCR amplification of the miRNA as shown in Supplementary Table 2. Reverse transcription of the extracted small RNA was carried out using a mixture of 1 μl of 5 μM room temperature (RT) primer, 4 μl total RNA, 1 μl dNTP Mix (10 mM each), 4 μl of 5x First-Strand Buffer, 2 μl of 0.1 M DTT, 1 μl of RNaseOUT (40 units/μl), 1 μl (200 units) of SuperScript-III reverse transcriptase. Reverse transcription was performed at 16 ˚C for 30 min followed by 42 ˚C for 30 min. PCR reaction (35 cycles) comprised 4 μl of template cDNA, 1 μl of 5 μM forward/reverse primers, 12.5 μl of HotStarTaq Master Mix (Qiagen) and 6 μl of PCR-grade water. PCR amplification was achieved through 40 cycles of denaturation (94 °C, 15 s) and annealing (60 °C, 45 s). After PCR amplification, products were run on a 1.5% agarose gel at 5 V/cm, stained in SYBR Gold for 45 min and destained in 1 × TAE buffer for 30 min before imaging. Imaging was performed using the MultiDoc-It™ Imaging System.

### Immunohistochemistry

After blocking in incubation buffer containing 0.1 M PBS, 1% BSA, 0.1% Tween-20, and 5% normal goat serum (for detection with rabbit Abs) or 5% normal rabbit serum (for detection with mouse Abs) for 40 min, sections were incubated with the primary antibodies overnight at 4 ˚C and secondary antibodies for 1 h at room temperature as per Supplementary Table 3. Specificity controls were carried out by incubating sections with rabbit or mouse IgG negative control antibodies.

### Primary antibody characterisation

1. Mouse monoclonal anti-p53 antibody (Abcam, catalogue no. ab26) recognises both mutant forms and wild-type human p53. The immunogen is gel-purified p53-beta-galactosidase fusion protein containing murine p53 from aa14-389 (derived from pSV53C cDNA clone). Mapping has demonstrated specificity to amino acids 213 to 217 on human p53 protein. The lysate from NIH/3T3 cells treated with 1 M doxorubicin for 24 h revealed a single band at 50 kDa corresponding to molecular weight of P53 [manufacturer’s technical information].
2. Rabbit polyclonal anti-P21 (Abcam, catalogue no. ab109199) detects a clean band at 21 kDa corresponding to p21, as well as a cross-reacting band at 26 kDa [manufacturer’s technical information]. The immunogen is a synthetic peptide conjugated to KLH derived from within residues 100 to the C-terminus of Human p21.
3. Rabbit polyclonal anti-Notch-1 (Abcam, catalogue no. ab8925) recognises an epitope (VLLSRKRRRQHGQC) that is only exposed after gamma secretase cleavage and is not accessible in the uncleaved form. The immunogen is human N-terminal sequence of the cleaved Notch-1 intracellular domain (amino acids 1755–1767).
4. Mouse monoclonal anti-tapa1 antibody (Abcam; cat. no. Ab79559) in Western blot recognises the low-molecular weight 26 kDa subunit of human tapa1. Immunogen is MOLT4 (human T-ALL cell line)
5. Monoclonal anti-mechanistic Target of Rapamycin (mTOR) antibody (Abcam; cat. no. Ab2732) was raised in Rabbit, which was immunised with a proprietary synthetic peptide within Human mTOR (aa 200–250). Western blotting shows a band at ≈ 250 kDa.
6. Mouse monoclonal anti-argonaute-2 (Ago2) antibody (Abcam; cat. no. Ab57113). The immunogen is recombinant fragment corresponding to human Ago2 aa483-859 and in western blots the antibody recognises a band of molecular weight 97 kDa.
7. The nestin antibody (Millipore, Bedford, MA; cat. no. MAB5326; clone 10C2) was raised in mouse by using a fusion protein. The antibody reacts specifically with human nestin and it recognises a major band at 200–220 kDa in western blot of human umbilical vein endothelial-cell (HUVEC) lysates (manufacturer’s technical information).
8. Polyclonal anti-Catenin-β1 antibody (Abcam; cat. no. Ab6302) was raised in Rabbit immunised with synthetic peptide (PGDSNQLAWFDTDL) conjugated to KLH and corresponding to amino acids 768–781 of Human β-Catenin. The antibody does not cross-react with a-catenin or γ-catenin (plakoglobin). In western blotting of extracts from Madin-Darby Bovine Kidney (MDBK) cultured cells the antibody recognises a single band of 94 kDa molecular weight.
9. Monoclonal anti-Oestrogen Receptor α1 (ESR1) antibody (Abcam; cat. no. Ab32063) was raised in Rabbit immunised with a proprietary synthetic peptide. In western blots the antibody recognises a single band at molecular weight of 60 kDa.
10. Mouse monoclonal anti-Progesterone Receptor (PGR) antibody (Abcam; cat. no. Ab2765). The antibody detects the B form of progesterone receptor and does not cross-react with oestrogen receptor or glucocorticoid receptor. The immunogenicity corresponds to chicken progesterone receptor and is purified using chick oviduct cytosol. Western blots detected a single band at 99 kDa.
11. Mouse monoclonal anti-Erbb2 antibody (Abcam; cat. no. Ab16901) was immunised with a synthetic peptide (TAENPEYLGLDVPV) which corresponds to C terminal amino acid 1242–1255 of human c-Erbb2.
12. Mouse monoclonal anti Ki-67 antibody (Dako, catalogue No. M72404) detects a nuclear protein Ki-67 antigen. The Ki-67 antigen is preferentially expressed during all active phases of the cell cycle (G1, S, G2 and M-phases), but it is absent in resting cells (G0-phase)^76^. Two isoforms of 345 and 395 kDa have been identified^77^. In western blotting of lysates of the multiple myeloma cell line, IM-9, the antibody labels bands of 345 and 395 kDa, identical to isoforms of Ki-67 protein (manufacturer’s technical information).

### **Autophagy inhibitors**

To inhibit the autophagic flux, Bafilomycin A1 (Sigma) and Chloroquine (Sigma) were applied at a final concentration of 10 nM and 50 μM, respectively. The cells were incubated with the inhibitors for 16 h and then processed for immunohistochemical staining as outlined before.

### **Western blotting**

Extracted proteins were separated re-separated by PAGE using gradient 5 to 12% minigels, transferred to 0.2-μm nitrocellulose membranes (Bio-Rad) and blocked for ≥ 2 h with 3% bovine serum albumin (Sigma) in 0.1 M Tris buffered salts solution pH 7.4 (TBS). Blotted antigens were incubated with a mouse monoclonal anti-HSP-70 (2 μg/ml, Abcam ab2897), rabbit monoclonal anti-Beclin-1(2 μg/ml, Abcam ab207612), rabbit polyclonal antiLC3β (1 μg/ml Abcam ab51520) and rabbit polyclonal anti-p62 (2 μg/ml Abcam ab91526), 0.05% Tween20/TBS for 2 h, washed and subsequently incubated with alkaline phosphatase (AP)-conjugated secondary antibody (goat-anti rabbit/mouse IgG, DAKO, Denmark) diluted 1:1500 in Tween20/TBS for 2 h. Bound antibody was visualised with AP substrate (BioRad).

### **Live-imaging analysis of pftLC3 autophagy reporter**

The autophagy reporter plasmid, ptfLC3, was a gift from Tamotsu Yoshimori (Addgene plasmid #21074). SKBR3 cells were electroporated with the reporter plasmid alone or in combination with miR4673 as described before. For live-imaging analysis cells were cultured in 6-well plates overnight and transferred into the live-imaging platform (Leica DMI6000B live cell imaging microscope). Phase contrast images were captured every 30 min for 16 h from multiple wells (20x magnification). To analyse mitotic activity, images were imported into FIJI (ImageJ) platform.

### Transmission electron microscopy

For TEM analysis, samples were fixed in Karnovsky’s fixative overnight at room temperature followed by post-fixation in OsO_4_ for 1 h. Preparations were dehydrated in graded alcohols and embedded in low viscosity resin (TAAB Laboratory and Microscopy, United Kingdom). Ultrathin sections were mounted on Pioloform/formvar coated slot grids, stained in uranyl acetate and lead citrate and examined in a Phillips CM120 BioTWIN electron microscope.

### Bioinformatics analysis

The expression profile of miR4673 in various human tissues was extracted from the miRmine database as per Supplementary Table 4. The raw output was analysed and a graph generated using the GraphPad software.

### **Quantification and statistical analysis**

SPSS statistical software (SPSS v.16, Chicago, Illinois, US) was used for the statistical analysis of data. The relative expression levels of genes of interest were compared using non-parametric Mann–Whitney *U*-test. In the present study, a *p*-value < 0.01 was considered as statistically significant.

## Electronic supplementary material

SUPPLEMENTAL TABLE 1

SuPPLEMENTAL TABLE 2

SuPPLEMENTAL TABLE 3

SUPPLEMENTAL TABLE 4
