# Supplementary material for: miR4673 improves fitness profile of neoplastic cells by induction of autophagy
Source: Cell Death Dis. 2018 Oct 19;9(11):1068. doi: 10.1038/s41419-018-1088-6 (PMC6195512; doi:10.1038/s41419-018-1088-6)
Supplement: Supplementary file 1 — SUPPLEMENTAL TABLE 1 [file 41419_2018_1088_MOESM1_ESM.docx]

**Supplementary Table 1. Transcript-specific PCR primers.**

| **Gene** | **Accession No.** | **Oligos** | **Primer sequence** | **Amplicon size (bps)** |
| --- | --- | --- | --- | --- |
| **rad51** | NM_002875.4 | F-primer  R-primer | TGCCAGCTTCCCATTGACCG  CCAGGACATCACTGCCAGAGAG | 130 |
| **ercc1** | NM_001983.3 | F-primer  R-primer | CTACCACAACCTGCACCCAGAC  GGGGATCTTTCACATCCACCTGG | 104 |
| **ppp1r13b** | NM_015316.2 | F-primer  R-primer | CCTGCTGGGGCTGTATCCAC  AAGTGGCTCCTGGTAGCTGG | 101 |
| **snai1** | NM_005985.3 | F-primer  R-primer | CGCTCTTTCCTCGTCAGGAA  GGCTGCTGGAAGGTAAACTCTGG | 95 |
| **Snai2** | NM_003068.4 | F-primer  R-primer | GCGAACTGGACACACATACAGTGA  GCGGTAGTCCACACAGTGATGG | 107 |
| **twist1** | NM_000474.3 | F-primer  R-primer | GGCCGGAGACCTAGATGTCATTG  CCCCACGCCCTGTTTCTTTGA | 133 |
| **xrcc3** | NM_001100119.1 | F-primer  R-primer | GCATCAACCAGGTGACAGAGGC  GGTCAGCCAGCAGTCTCACC | 135 |
| **brca1** | NM_007294.3 | F-primer  R-primer | CAGCTTGACACAGGTTTGGAGTATGC  GGCACGGTTTCTGTAGCCCAT | 123 |
| **brca2** | NM_000059.3 | F-primer  R-primer | TTGTGAAGGGTCGTCAGACACC  GCACAGTAGAACTAAGGGTGGGTG | 117 |
| **ccnd1-short isoform** | NM_053056.2 (ENST00000227507.2) | F-primer  R-primer | CACCTGGATGCTGGAGATGTGAAG  AGGCGGTAGTAGGACAGGAAGTTG | 128 |
| **ccnd1-long isoform** | ENST00000536559.1 | F-primer  R-primer | CTGCTGCAAATGGAGCTGCTC  CTGTTTGTTCTCCTCCGCCTCTG | 120 |
| **cdk1** | NM_002596.3 | F-primer  R-primer | CCGAGAGGTGTCTCTGCTGAAG  CAGGTACTCAAACACCAGGGTGAG | 103 |
| **notch1** | NM_017617.4 | F-primer  R-primer | GCATCTGTGCCAGTACGATGTGG  CCGTGTACCCTTCCGTGCA | 113 |
| **xbp1-short isoform** | NM_001079539.1 | F-primer  R-primer | CTGAGTCCGAAGCAGGTGCAG  ATGCCCAACAGGATATCAGACTCTGA | 109 |
| **xbp1-long isoform** | NM_005080.3 | F-primer  R-primer | GCAGCACTCAGACTACGTGCA  ATGCCCAACAGGATATCAGACTCTGA | 127 |
| **ctnbb1-short isoform** | NM_001330729.1 | F-primer  R-primer | TGGCTACTCAAGCTGATTTGATGGAGT  ACTCCATCAAATCAGCTTGAGTAGCCA | 125 |
| **ctnbb1-long isoform** | NM_001098209.1 | F-primer  R-primer | GAAGGTGTGGCGACATATGCAGC  AGATCAGCAGTCTCATTCCAAGCCA | 143 |
