# Supplementary material for: miR4673 improves fitness profile of neoplastic cells by induction of autophagy
Source: Cell Death Dis. 2018 Oct 19;9(11):1068. doi: 10.1038/s41419-018-1088-6 (PMC6195512; doi:10.1038/s41419-018-1088-6)
Supplement: Supplementary file 2 — SuPPLEMENTAL TABLE 2 [file 41419_2018_1088_MOESM2_ESM.docx]

**Supplementary Table 2. Primers used in stem–loop RT–PCR.**

| **Primer** | **Utility** | **Sequence** |
| --- | --- | --- |
| SL-RT | RT primer | GTCGTATCCAGTGCAGGGTCCGAGGTATTCGCACTGGATACGACGCCGCA |
| SL-F | Forward primer | CACGCAGTCCAGGCAGGA |
| SL-R | Reverse primer | CCAGTGCAGGGTCCGAGGTA |
