# Supplementary material for: miR4673 improves fitness profile of neoplastic cells by induction of autophagy
Source: Cell Death Dis. 2018 Oct 19;9(11):1068. doi: 10.1038/s41419-018-1088-6 (PMC6195512; doi:10.1038/s41419-018-1088-6)
Supplement: Supplementary file 3 — SuPPLEMENTAL TABLE 3 [file 41419_2018_1088_MOESM3_ESM.docx]

**Supplementary Table 3. Primary and secondary antibodies utilized in the current study.**

| **Antibody** | **Immunogen** | **Details** | **Dilution** |
| --- | --- | --- | --- |
| p53 | p53-β-galactosidase fusion protein | Abcam, mouse monoclonal, Ab26 | 1:200 (5μg/ml) |
| p21 | Synthetic peptide conjugated to KLH | Abcam, rabbit polyclonal, Ab18209 | 1:100 (5μg/ml) |
| Notch1 | Synthetic peptide from C-terminal of Human Notch-1 | Abcam, rabbit polyclonal, Ab27526 | 1:50 (4μg/ml) |
| Tapa1 | Human T-All cell line | Abcam, mouse monoclonal, Ab79559 | 1:200 (5μg/ml) |
| mTOR | Synthetic peptide within Human mTOR | Abcam, rabbit monoclonal, Ab2732 | 1:200 (5μg/ml) |
| Ago2 | Recombinant fragment corresponding to mTOR | Abcam, mouse monoclonal, Ab57113 | 1:100 5μg/ml |
| Nestin | AA conjugated to glutathione S transferase | Abcam, mouse monoclonal, Ab22035 | 1:200 (5μg/ml) |
| Cateninβ1 | Synthetic peptide conjugated to KLH | Abcam, rabbit polyclonal, Ab6302 | 1:1 000 (3.18μg/ml) |
| ESR1 | Synthetic peptide within Human Estrogen Receptor 2 | Abcam, rabbit monoclonal, Ab32063 | 1:500 4.9μg/ml |
| PGR | Immunogen Type corresponding to Chicken PGR | Abcam, mouse monoclonal, Ab2765 | 1:200 (5μg/ml) |
| Erbb2 | Synthetic peptide corresponding to c-Erbb2 | Abcam, mouse monoclonal, Ab16901 | 1:200 (5μg/ml) |
| Ki67 |  | Dako, Mouse monoclonal, M72404 | 1:200 (5μg/ml) |
