# Supplementary material for: miR4673 improves fitness profile of neoplastic cells by induction of autophagy
Source: Cell Death Dis. 2018 Oct 19;9(11):1068. doi: 10.1038/s41419-018-1088-6 (PMC6195512; doi:10.1038/s41419-018-1088-6)
Supplement: Supplementary file 4 — SUPPLEMENTAL TABLE 4 [file 41419_2018_1088_MOESM4_ESM.docx]

**Supplementary Table 4. List of tissues presented in Figure 1A of the main text.**

| **Tissue** | **Description** | **Disease** | **Log_2_(RPM)** |
| --- | --- | --- | --- |
| Breast | Tumor serum | Breast cancer | 95.1 |
| Breast | Serum from healthy woman control | Normal | 46.2 |
| Bladder | Sample#6_bladder cancer_UC_G3_pT2_65yo_male | Bladder cancer | 1.9 |
| Plasma | CFS3 | Chronic fatigue syndrome | 1.6 |
| Plasma | NF4 | Non-fatigued | 0.9 |
| Brain | ETMR4-rna | Embryonal brain tumor | 0.8 |
| Brain | ETMR3-rna | Embryonal brain tumor | 0.6 |
| Lung | Adenocarcinoma with bone metastasis | Cancer | 0.4 |
| Blood | hPBMCs exposed to F. tularensis Schu S4 (30, 60, and 120 min) |  | 0.4 |
| Bladder | Sample#10_bladder cancer_UC_G3_pT2_83yo_male | Bladder cancer | 0.3 |
| Nasopharynx | FFPE-Ctrl1 |  | 0.3 |
| Bladder | Sample#7_bladder cancer_UC>SCC_G3_pT3_76yo_male | Bladder cancer | 0.2 |
| Brain | ETMR5-rna | Embryonal brain tumor | 0.2 |
| Lung | Adenocarcinoma without bone metastasis | Cancer | 0.1 |
| Bladder | Sample#2_normal bladder epithelia | Normal | 0.1 |
| Bladder | Sample#3_normal bladder epithelia | Normal | 0.1 |
| Bladder | Sample#5_normal bladder epithelia | Normal | 0.1 |
| Bladder | Sample#8_bladder cancer_UC_G2_pT2_91yo_male | Bladder cancer | 0.1 |
| Plasma | CFS1 | Chronic fatigue syndrome | 0.1 |
| Blood | BGI-150p2 |  | 0.1 |
